# Identification of genes for controlling swine adipose deposition by integrating transcriptome, whole-genome resequencing, and quantitative trait loci data

**DOI:** 10.1038/srep23219

**Published:** 2016-03-21

**Authors:** Kai Xing, Feng Zhu, LiWei Zhai, ShaoKang Chen, Zhen Tan, YangYang Sun, ZhuoCheng Hou, ChuDuan Wang

**Affiliations:** 1 National Engineering Laboratory for Animal Breeding and MOA Key Laboratory of Animal Genetics and Breeding, Department of Animal Genetics and Breeding, China Agricultural University, Beijing 100193, China; 2 Beijing General Station of Animal Husbandry, Beijing 100125, China

## Abstract

Backfat thickness is strongly associated with meat quality, fattening efficiency, reproductive performance, and immunity in pigs. Fat storage and fatty acid synthesis mainly occur in adipose tissue. Therefore, we used a high-throughput massively parallel sequencing approach to identify transcriptomes in adipose tissue, and whole-genome differences from three full-sibling pairs of pigs with opposite (high and low) backfat thickness phenotypes. We obtained an average of 38.69 million reads for six samples, 78.68% of which were annotated in the reference genome. Eighty-nine overlapping differentially expressed genes were identified among the three pair comparisons. Whole-genome resequencing also detected multiple genetic variations between the pools of DNA from the two groups. Compared with the animal quantitative trait loci (QTL) database, 20 differentially expressed genes were matched to the QTLs associated with fatness in pigs. Our technique of integrating transcriptome, whole-genome resequencing, and QTL database information provided a rich source of important differentially expressed genes and variations. Associate analysis between selected SNPs and backfat thickness revealed that two SNPs and one haplotype of *ME1* significantly affected fat deposition in pigs. Moreover, genetic analysis confirmed that variations in the differentially expressed genes may affect fat deposition.

The consumption of pig meat products is increasing worldwide^1^. Fatness traits are very important in pork production because they are closely related to meat quality, fattening efficiency, reproductive performance, and immunity. Indeed, backfat thickness has a strong influence on lean meat percentage, intramuscular fat content, growth rate, feed conversion rate, and reproductive performance^2^,^3^, so fat deposition plays an important role in pig production.

The fat content of meat is also an important consumer choice because of the association of unhealthy food with obesity^4^, and the public health issue of fat deposition in humans. Obesity is not only a risk in itself, but also contributes to its comorbidities such as type 2 diabetes, cardiovascular disease, and certain forms of cancer^5^. Pigs are an important animal model for the study of the genetic basis of obesity, particularly when compared with rodent models^6^, because of their similarity to humans in terms of genetics, body size, and other physiological and anatomical features, including their innate tendency to overconsume food.

Adipose tissue is a complex, essential, highly active metabolic and endocrine organ that acts as a fat storage depot. It is the principal organ involved in circulating free fatty acid (FA) and regulating lipid metabolism. Adipose tissue is also the site of *de novo* FA synthesis in pigs^7^. It produces and releases adipokines such as tumour necrosis factor α, peptide hormones such as leptin, adiponectin, estrogen, and resistin, and lipid hormones (lipokines) such as palmitoleate^8^. Adipokines are involved in the maintenance of metabolic homeostasis and play an important role in the development of diseases associated with obesity, including insulin resistance, inflammation, hypertension, cardiovascular disease, and metabolic disorders^9^. Thus, the transcriptome analysis of adipose tissue may improve our understanding of its distinct features as well as swine adipose deposition.

Recent studies have focused on the transcriptome of porcine adipose tissue, focusing on differences in breeding^10^,^11^, phenotype^12^,^13^, developmental period^14^, and storage position^15^. However, current reports used limited controls of genetic backgrounds for detecting genes influencing fat deposition. Previously, we used RNA-seq to analyse the livers and adipose tissue of six Songliao black pigs classified in two phenotypically extreme groups for backfat thickness^16^,^17^. The Landrace pigs, strongly artificially selected for backfat thickness, are less able to deposit fat compared with Songliao black pigs.

In the present study, we applied RNA-seq to identify differences in adipose tissue transcriptomes from three pairs of full-sibling (full-sib) Danish Landrace sows with opposite backfat thickness phenotypes. Meanwhile, whole-genome resequencing was used to investigate the genetic basis for variations between the two groups with high/low backfat thickness. Our experimental design is shown in Fig. 1. Finally, we integrated RNA-seq, whole-genome resequencing data, and information from the animal quantitative trait loci (QTL) database to identify potential key genes and variations influencing swine adipose deposition.

## Results

### Animal phenotypes

A Landrace pig resource population (average age, 186 days; average live weight, 93.38 kg) was used in this study. Three full-sib Landrace pairs were selected from the pool of 132 pigs with an average backfat thickness of 5.76 ± 1.75 mm. Three of the pigs with high backfat thickness (8.70 ± 1.05 mm) formed the high group (LH) and three with low backfat thickness (3.80 ± 0 mm) formed the low group (LL). Because backfat thickness is a good indicator, other fatness traits, such as carcass backfat thickness and kidney fat weight, showed significant differences between the two groups (Table 1).

### Analysis of RNA-seq data

Six cDNA libraries from six samples of backfat tissues (three from LH and three from LL) were sequenced using HiSeq 2000. The total number of PE reads ranged from 37.19 million to 40.08 million (average, 38.69 million) from each cDNA library. The total number of reads for each sample and the percentage of reads mapped to reference genome sequences are shown in Supplementary Table S1. An average 78.68% of total reads were aligned to reference genome sequences, while averages of 73.31%, 2.16%, 11.71%, and 5.66% aligned reads were within coding sequences (CDS), 5′ untranslated regions (UTRs), 3′UTRs, and introns, respectively.

### Differential gene expression analysis

**Figure 2:**
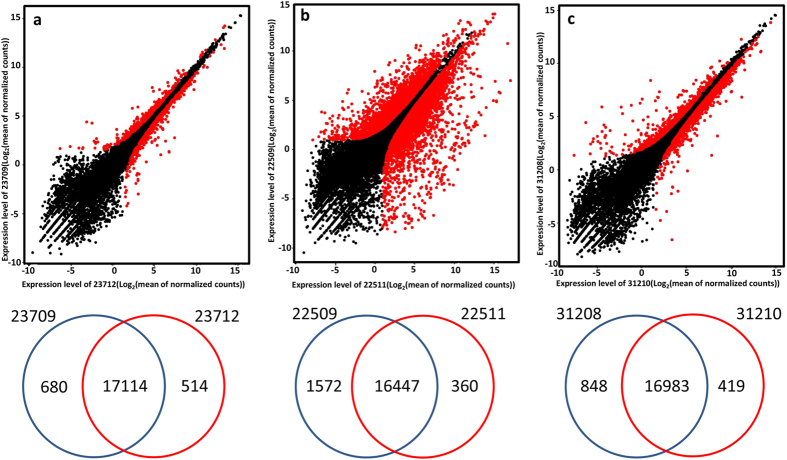
Scatter diagrams representing gene expression of full-sibling pig comparisons with extreme levels of backfat thicknesses. Gene expression levels for each individual of each of three pairs of pigs: 23709 and 23712 (**a**), 22509 and 22511 (**b**), and 31208 and 31210 (**c**) are shown. Red points represent differentially expressed genes. Venn diagrams show the total number of expressed genes in each individual. The number of common genes is shown in the overlapping segments.

The total number of expressed genes in adipose tissue was similar between samples (17,212–18,547). Most genes were expressed in both of the full-sib individuals being compared, so the major fraction of adipose transcriptome appears to be conserved. No significant difference was observed in the mean normalized read count between each full-sib pair with high and low backfat thickness (Fig. 2).

A total of 2,052, 7,362, and 781 differentially expressed genes (DEGs) were identified between each of the three full-sib pairs. Forty-eight DEGs were found to be highly expressed in the high backfat thickness group, while 41 DEGs were highly expressed in the low backfat thickness group (Supplementary Fig. S1). A total of 27 DEGs were related to lipid synthesis, transport, and metabolism (Supplementary Table S2). Heat maps illustrate the common DEGs identified in adipose tissue of pigs with high and low backfat thickness (Supplementary Fig. S2).

### Functional annotation clustering of DEGs

After converting 89 DEGs to human orthologs, 71 were assigned to a specific functional group based on information from Database for Annotation, Visualization and Integrated Discovery (DAVID). GO functional enrichment analysis including molecular functions (MFs), biological processes (BPs), and cellular components (CCS) of up-regulated DEGs indicated that they were primarily enriched in categories related to oxidation reduction (e.g., oxidation reduction and oxidoreductase activity), metabolic processes (e.g., generation of precursor metabolites and energy, alcohol catabolic process, glycolysis, and glucose catabolic process), transport processes (e.g., electron transport chain and hydrogen ion transmembrane transporter activity), and biosynthetic processes (e.g., acetyl-CoA biosynthesis from pyruvate and the coenzyme biosynthetic process).

The enriched GO metabolic processes of down-regulated DEGs include immune responses (e.g., opsonization, response to protein stimulus, and the immune effector process), transcriptional regulation (e.g., negative regulation of transcription, negative regulation of RNA metabolic processes, and transcription repressor activity), and transport processes (e.g., carbohydrate binding, calcium ion binding, and calcium-dependent protein binding) (Supplementary Fig. S2).

A total of 33 DEGs were mapped to Kyoto Encyclopedia of Genes and Genomes (KEGG) pathways. KEGG pathway analysis identified pyruvate metabolism, oxidative phosphorylation, glycolysis/gluconeogenesis, and the citrate cycle (TCA cycle) as the dominant up-regulated pathways that play regulatory roles in metabolism. Other pathway categories including cardiac muscle contraction and the transforming growth factor-β signaling pathway were also enriched (Table 2).

**Figure 3:**
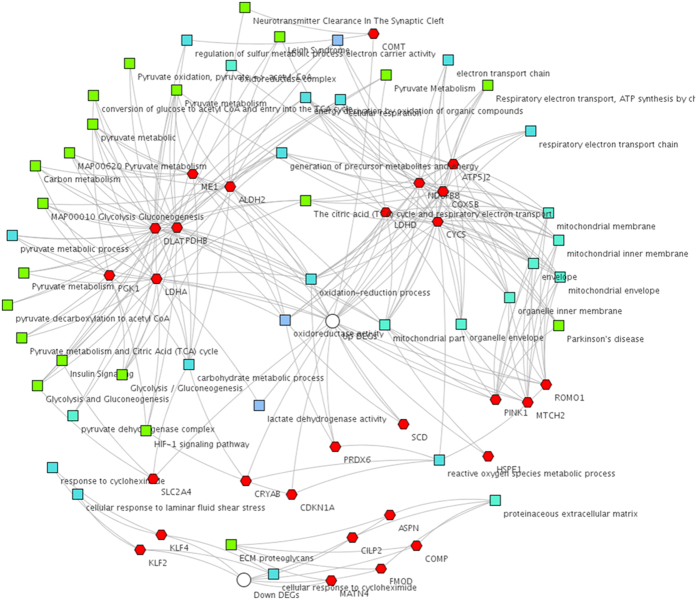
Relationship between DEGs, pathways, and GO terms. Functional association analysis performed by ToppCluster based on pathway networks showing enriched terms from Gene Ontology and pathways. The top part of the figure depicts significant enrichments for up-regulated DEGs in LH; the lower part depicts significant enrichments for down-regulated DEGs in LH. Red hexagon: DEG; green square: pathway; blue square: biological processes; grey square: molecular function.

Figure 3 shows the graphical network of ToppCluster results. BPs related to metabolism, immunity, muscle cell differentiation, organ morphogenesis, and oxidation and reduction were detected. Abstracted network analysis revealed a distinct functional separation between the DEGs and GO terms, while those genes related to metabolic and transport processes in adipose tissue that could confer phenotypes were interlinked to aspects of lipid synthesis.

### Analysis of genetic variations and functional annotations

Pooled genomic DNA from three Landrace pigs with high/low backfat thickness was sequenced using Hiseq 2000. The resulting reads yielded approximately 10-fold (25G data) coverage for each pool and mapped to a reference pig genome (Sscrofa10.2). Using GATK, putative SNPs and indels were identified by comparing the aligned reads to the reference assembly. More than 3,591,983 genetic variations, including 3,285,127 SNPs and 306,856 indels, were detected for LH and LL genomes. Most of these were located between genes or within introns (Table 3).

Because backfat thickness phenotypes diverged between the two groups, 1,483,491 SNPs and 306,856 indels specific to one group were focused on. The SNPs only present in LH/LL were annotated to 16,339/17,158 genes, while 11,408/10,108 genes had indels in LH/LL, respectively. Taking the above information into account, a total of 1,456 and 1,243 genes with NS/CI were found in LH and LL groups, respectively.

After conversion to homologous human genes, those genes with NS/CI underwent functional annotation clustering analysis to identify MF, BP, CC, and KEGG pathways in DAVID. The genes with variants (NS/CI), which were only shown in one group, were enriched in numerous pathways, including synthesis, metabolism, oxidation–reduction, enzyme activity, molecular transport, and binding (Supplementary Fig. S3). Interestingly, the olfactory transduction pathway was identified as the most significant in both LH-specific mutated variants and LL-specific mutated variants, involving 75/72 genes in LH/LL. Through GO and pathway analysis, 38 genes were shown to be involved in lipid-related processes such as lipid biosynthesis, transport, localization, and binding, FA metabolism, and the regulation of lipid kinase activity.

### DEGs compared with the QTL database and previous reports

From the animal QTL database, 951 QTLs were selected for the present study, and 20 DEGs were matched to the chosen QTLs, representing candidate genes affecting fat deposition. We also identified 21 DEGs that were common to earlier studies, and which may represent additional candidate genes (Supplementary Table S3).

### Mutations in DEGs supported by whole-genome resequencing data

A large number of SNPs and indels were only detected in DEGs in adipose tissue between LH and LL groups (Supplementary Table S4). Key variations, such as frame shifts, splice site mutations, UTR and non-synonymous coding lesions, were identified in genes regulating lipid processes between LH and LL groups. Additionally, many SNPs were detected in important regions of the key DEGs. For instance, an SNP in *CYP21A2* and a non-synonymous SNP in the coding region of *FCN2* were predicted to alter the protein sequence. A key gene in the biosynthesis of unsaturated FAs, *SCD*, was shown to contain an SNP in the 5′UTR, while other UTR SNPs were identified in genes such as *UQCRFS1*, *ME1*, and *PRDX6*. Abundant variation in introns, and regions 5 kb upstream and downstream of DEGs was also observed.

### SNP associate analysis with backfat thickness

Based on the above information, we selected SNPs for an association study with backfat thickness. The effects of the analysed SNPs on backfat thickness traits are presented in Supplementary Table S5. Two polymorphisms (1-93240107 and 1-93240683) in *ME1* were significantly associated with backfat thickness (*p* < 0.05). At position 1-93240107, pigs with genotype CA had higher backfat thickness than those with genotypes CC and AA. Moreover, at position 1-93240683, pigs with genotype GG had higher backfat thickness than those with genotypes GT and TT (Table 4). No other SNPs were significantly associated with fat deposition traits.

Haploview software detected one haplotype block, composed of the two *ME1* SNPs (Supplementary Fig. S4), reflecting two kinds of haplotypes (H1:AT; H2:CG) with a frequency of 27.11% and 72.89% respectively. These were shown to be significantly associated with backfat thickness (*p* = 0.019). The genotypes of haplotypes effecting backfat thickness indicate the existence of significant differences among groups (Table 4). Moreover, the findings of single-locus and haplotype association analyses were consistent with each other.

## Discussion

In our study, we presented the systematic transcriptome profiling of adipose tissue from three full-sib pairs of pigs with extreme backfat thickness using high-throughput RNA-seq technology. Compared with previous studies^10^,^11^,^13^, using three full-sib pairs of pigs could reduce false-positive results caused by genetic background noise and the number of replicates. DEGs were also detected for three pigs with similar backfat phenotypes, which were considered replicates, and these DEGs differed from those revealed by matched pair experiments.

**Figure 4:**
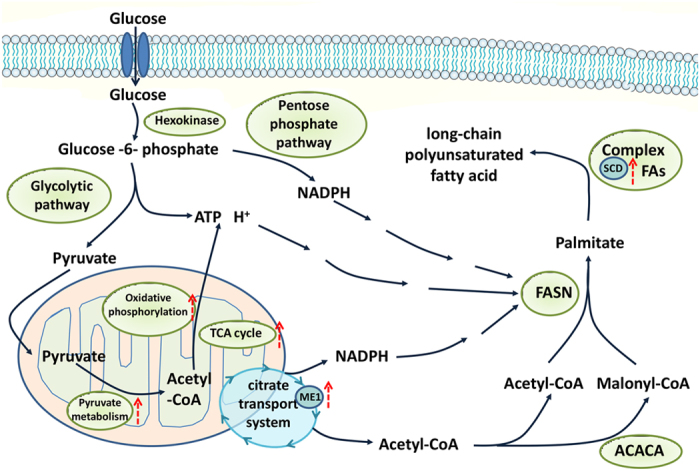
Schematic of potential role of up-regulated genes and pathways in *de novo* fatty acid synthesis.

Adipose tissue is the primary location of *de novo* FA synthesis in pigs^7^. We identified several genes and pathways involved in FA synthesis that were up-regulated in the LH compared with the LL group (Fig. 4). As an example, the rate-limiting enzyme stearoyl-CoA desaturase (encoded by *SCD*) plays a role in the biosynthesis of unsaturated FA. *SCD* was more highly expressed in LH than LL, which might explain the observed higher backfat thickness in LH^18^. Previous reports have demonstrated that *SCD* polymorphisms are related to fatty traits^19^, so our identification of an SNP in the *SCD* 5′UTR suggests that this a good candidate locus for fat deposition.

Malic enzyme (encoded by *ME1*) is involved in the TCA cycle to supply NADPH and to transport acetyl-CoA from mitochondria to the cytosol for the biosynthesis of FAs^20^. Its functions in glucose metabolism also contribute to the initial steps of lipogenesis^12^. *ME1* mRNA was more abundant in the LH compared with the LL group in our study, which is consistent with the biological function of this gene^21^,^22^,^23^. Moreover, we detected *ME1* variations, including six SNPs in the 5′UTR, which may be relevant to the deposition of fat.

The first step in FA synthesis is the conversion of acetyl-CoA to malonyl-CoA. In the chondriosome, acetyl-CoA is produced from the metabolism of pyruvate, which is enriched in KEGG pathways, as shown in our study. *ATP5J2*, *COX5B*, and *COX6B* encode proteins involved in adenosine triphosphate (ATP) synthesis, and were all found at a greater abundance in the LH group. Meanwhile, two representative pathways of glucose metabolism, oxidative phosphorylation and the TCA cycle, were also more highly expressed in the LH group. This suggests that more energy would be supplied for the citrate transport system and FA synthesis.

FA β-oxidation (FAO) plays a pivotal role in energy homoeostasis. Carnitine palmitoyltransferase I (encoded by *CPT1)*, which converts acyl-CoA to acylcarnitine, is the key rate-limiting enzyme in FAO, and is regulated by its inhibitor malonyl-CoA^24^. The cellular energy status is one of the regulators of malonyl-CoA production. At times of low energy, ATP levels fall and adenosine monophosphate levels (AMP) rise, resulting in activation of AMP-activated protein kinase, which initiates a signaling cascade to restore cellular energy levels^25^. In our study, the observed high expression levels of *CPT1A* in the LL group indicate that more FAO occurs in the low backfat thickness group.

The cytochrome P450 superfamily of genes (*CYP*) encodes enzymes that convert saturated and unsaturated FAs into small molecules of widely different chemical structures. *CYP21A2* encodes a CYP protein that hydroxylates steroids at position 21 and is required for the synthesis of steroid hormones including cortisol and aldosterone.

*NDUFB8* and *LDH* play important roles in energy metabolism, and had higher expression levels in the LH group. *NDUFB8* encodes the NADH dehydrogenase (ubiquinone) 1 beta subcomplex, which is involved in the electron transport chain/energy metabolism, and was previously shown to be up-regulated in response to elevated plasma low-density lipoprotein and triglyceride levels^26^. Polymorphisms of *NDUFB8* were also associated with circulating levels of FAs^27^. Lactate dehydrogenase catalyses the conversion of pyruvate to lactate, which releases energy. It also plays a role in controlling lactate formation and turnover regulation^28^. LDHA was previously shown to be significantly associated with backfat thickness^28^ and was mapped to SSC 2p, which is in a QTL region for fatness traits, including average backfat thickness and fat percentage^29^.

Inhibitor of DNA binding (ID) proteins are helix-loop-helix transcription factors that regulate the proliferation and differentiation of cells, and are highly expressed in adipose tissue. Previously, loss of Id1 in mice increased the proportion of lipid as an energy source for increased energy expenditure and reduced fat mass^30^. Glucose uptake by skeletal muscle and brown adipose tissue was also increased, suggesting that these tissues demonstrate increased glucose metabolism and thermogenesis^31^. In our study, both *Id1* and *Id2* were down-regulated in the LH group, but further study is needed to understand their role in lipid metabolism.

In the present study, we selected 14 SNPs from five candidate genes (*SCD*, *ME1*, *FASN*, *CRYAB*, and *PCK1*) by integrating transcriptome and whole-genome resequencing; two SNPs in *ME1* were significantly associated with backfat thickness (*p* < 0.05). QTL on porcine chromosome 1 have previously been identified as affecting backfat thickness; however, no QTL in *ME1* had a significant influence on body composition or meat quality in a Landrace population in this region^32^. *ME1* genotypes are associated with fatness and meat quality traits in Landrace pigs. More specifically, *ME1* showed significant associations with backfat thickness at 171 days, as well as muscle pH, redness, and yellowness^33^. *ME1* polymorphisms were also found to significantly affect leanness in turkeys^34^. Our results are consistent with these prior findings, and reveal an important role for *ME1* in fat metabolism. Transcriptome and whole-genome resequencing data revealed abundant genetic variations that are considered candidate loci effecting fat deposition. However, SNPs from other selected genes were not associated with backfat thickness. This could be explained by: (1) the small number of individuals in the reference population; (2) the genotypes of chosen SNPs not being in Hardy–Weinberg equilibrium; and (3) the small number of SNPs chosen from each gene for the association study. More studies are needed to determine their specific functions.

In conclusion, this study describes a global view of the adipose tissue (backfat) transcriptome and genome of two Landrace pig groups with diverging backfat thicknesses. A comparison of the transcriptomes between each of the three pairs of full-sib pigs identified 2,052, 7,362, and 781 DEGs, of which 89 overlapped among the three pairs. These genes encode proteins with functions in FA synthesis and lipid metabolism. Genes and pathways involved in *de novo* FA synthesis such as *SCD*, *ME1*, pyruvate metabolism, oxidative phosphorylation, and the TCA cycle were up-regulated in the high backfat thickness group, while those related to lipid metabolism were expressed at greater abundance in the low backfat thickness group. By comparison with the reference genome sequence, 2,602,847 SNPs and 191,609 indels were identified in the high backfat thickness group, and 2,483,855 and 172,693, respectively, in the low backfat thickness group. Compared with the animal QTL database, 21 DEGs were matched to the QTLs associated with fatness in pigs. SNP associate analysis with backfat thickness identified two *ME1* SNPs that appear to significantly affect fat deposition traits of pigs. One haplotype block, composed of both SNPs and reflecting two kinds of haplotypes (H1:AT; H2:CG), was also significantly associated with backfat thickness. Taking these data into account, our research identified potential candidate genes associated with fat deposition traits in Landrace pigs.

## Methods

### Animals, phenotypes, and samples

The Landrace population of female pigs was provided by the Tianjin Ninghe primary pig breeding farm (Ninghe, China). Pigs were housed in standard conditions with natural, uncontrolled room temperature and light. Animals were fed three times a day and had access to water *ad libitum*.

A total of 132 female Landrace pigs were measured using real-time B-mode ultrasonography with an HS1500 convex scanner (Honda Electronics, Toyohashi, Japan) for backfat thickness between the 3rd and 4th last ribs to choose pairs of animals with divergent backfat phenotypes. Age, weight, backfat thickness, and pedigree information were available. Selection methods and standards included two prerequisites: LH group individuals of each pair had to have at least twice the backfat thickness of the other member of the pair in the LL group, and pairs of pigs with divergent backfat thicknesses were full-sibs. Based on our criteria, three pairs of full-sib pigs were chosen with each pair having opposite backfat thickness phenotypes.

All selected pigs were stunned with a captive bolt, exsanguinated, and slaughtered in a commercial abattoir (Beijing Huadu Sunshine Food Co., Ltd., Beijing, China). The chosen pigs were slaughtered according to pig slaughtering guidelines (GB/T 17236–2008) endorsed by the General Administration of Quality Supervision, Inspection and Quarantine of the People’s Republic of China and the Standardization Administration of the People’s Republic of China. This study was approved by the Animal Welfare Committee of China Agricultural University (permit number: DK996). All efforts were made to minimize animal suffering during the study. Subcutaneous backfat adipose tissue between the 3rd and 4th last ribs was isolated aseptically and frozen in liquid nitrogen immediately after slaughter until required for RNA isolation. Liver tissue was collected and stored at −20 °C until required for DNA extraction.

### RNA isolation, library preparation, and sequencing

Total RNA from subcutaneous adipose tissue was isolated using the total RNA extraction kit (Bioteke, Beijing, China) according to the manufacturer’s recommendations. RNA quality was assessed by electrophoresis on a 1% agarose gel and an Agilent 2100 Bioanalyzer (Agilent, Santa Clara, CA). Paired-end (PE) libraries were prepared according to the Illumina paired-end library preparation protocol (Illumina, San Diego, CA). PE libraries were sequenced on an Illumina Hiseq 2000 sequencing system to generate 2 × 90 PE reads.

### DNA extraction, library construction, and sequencing

Genomic DNA was extracted from liver samples using the TIANamp Genomic DNA Kit (Tiangen Biotech, Beijing, China). Purified DNA was assessed for purity and quality by a NanoDrop2000 spectrophotometer (Thermo Scientific, Wilmington, DE), 1% gel electrophoresis, and an Agilent 2100 Bioanalyzer (Agilent). Two pools of genomic DNA (LH and LL, three samples from each) were used to create PE libraries according to the manufacturer’s instructions (Illumina). Purity and yield were checked using the 2100 Bioanalyzer (Agilent). PE sequencing was performed on a Hiseq 2000 sequencer and high-quality reads of 2 × 110 bp PE were obtained for each group.

### Mapping, assembly, and annotation of RNA-seq reads

Data analysis included an initial quality control and filtering step. Raw reads from each sequencing library were assessed using FASTQC (http://www.bioinformatics.babraham.ac.uk/projects/fastqc/) to remove adaptor sequences, reads with unknown sequences “N”, and low-quality sequences (the percentage of low-quality bases with a threshold quality score <20). Clean reads were mapped onto the reference pig genome (Ensembl Genes v67) using TopHat v2.0.1 software^35^ with default parameters. The number of reads mapping to exons, introns, and intergenic positions in the genome was calculated using BED Tools^36^. The easyRNASeq package in R^37^ was used to count the number of annotated clean reads for each gene.

### Differential gene expression analysis

The number of annotated clean reads for each gene was calculated and normalized using the trimmed mean of M-values (TMM) normalization method^38^. This is a simple and effective technique for estimating relative RNA expression level from RNA-seq data to lower false-positive rates and provide higher power to detect true differences. DEG analysis was performed to contrast differences in gene expression between full-sib samples. DEG analysis with TMM values was conducted using R package NOISeq^39^, which is a nonparametric approach for the identification of DEGs. The NOISeq method takes into account the noise distribution from actual data, better adapts to the size of the data set, and is more effective at controlling the rate of false discoveries^40^. To measure expression level changes between two conditions, two statistics were considered: contrasting fold-change differences (M) and absolute expression differences (D). The fold-change for features with low read counts can be misleading, as can the difference in expression between two conditions for the high counts. A threshold of 0.8 was used for this probability, meaning that the gene was four times more likely to be differentially expressed than non-differentially expressed, with an adjusted *p*-value threshold of 0.001. The threshold value of fold-change (FC) was 1.5. Intersections of the DEGs between pigs in each pair were followed by functional annotation clustering as described below to limit false positives.

### DEG functional annotation clustering

DEGs were converted to human orthologs and underwent functional enrichment analysis^15^. Gene Ontology (GO) and KEGG pathway enrichment analysis^41^ with features corresponding to DEGs at the intersection of DEGs between pigs of each pair was performed using DAVID v6.7 (http://david.abcc.ncifcrf.gov/)^42^.GO terms and pathways with *p*-values < 0.05 were deemed to be significant. ToppCluster^43^ identified biological themes in data sets involving numerous sets of genes, regulatory networks, and systems biology-based dissections of biological states. A visualized network enriched from pathway and DEG lists was analysed. A ToppCluster *p*-value cutoff of 0.05 was selected.

### Short-read alignment, variant calling, and annotation of whole-genome resequencing data

PE 110 bp reads from two groups (LH and LL) were mapped to the reference pig genome (Sscrofa10.2) using Burrows–Wheeler Alignment tool (BWA) software with default parameters^44^. The reads mapped to multiple chromosomal positions were used for single nucleotide variant (SNV) and indel variants calling, while unmapped reads were discarded. The coverage and accuracy of SNV and indel calling were evaluated using BWA by sequence read depth. We first filtered out reads with a mapping quality score <20. After quality score recalibration, local indel realignment and the removal of duplicates from alignments, SNV and indel calling was performed using the Genome Analysis Toolkit (GATK)^45^. The genetic variants identified in two groups (LH and BL) were identified using Ensembl gene sets (30,711 genes; available from the Ensembl BioMart site [http://www.ensembl.org/biomart/]). We selected non-synonymous (NS) single nucleotide polymorphisms (SNPs) and coding indels (CI) as potential variants affecting the phenotype. Unique NS/CI variants were detected in each group by their genomic position for downstream analysis.

### Functional enrichment of genes with non-synonymous/coding indels

We determined genes that overlapped partially or completely with the NS/CI for each group. These candidate genes underwent functional enrichment analysis within GO and KEGG pathway terms using DAVID v6.7. The threshold value of significance was <0.05 for GO terms and KEGG pathways.

### DEG comparison with the animal QTL database and previous transcriptome information

To identify candidate genes associated with porcine fat deposition, we integrated DEGs and QTLs for fatness traits collected from the animal QTL database (http://www.animalgenome.org/QTLdb)^46^ by comparing their chromosome positions. This database houses all publicly available QTL data on livestock and includes 11,610 QTLs, of which 1,741 are associated with fatness in pigs. We also took into account the size of the published QTL region. Only those with a confidence interval less than 5 Mb and related to fatness were considered as an available QTL, so a total of 951 QTLs were chosen in this study. Because several previous studies have analysed backfat transcriptomes in different breeding groups with diverging fat deposition capacities^10^,^11^ or fatty/lean phenotypes^13^,^16^, the DEGs in our analyses were also compared with these reports.

### SNP association analysis with backfat thickness

Based on the above information, some SNPs were selected for further analysis because they were associated with live backfat thickness. These are listed in Supplementary Table S6, and were selected, based on the following criteria: i) located in the DEGs between LH and LL, which play important roles in fat deposition; ii) detected in only LH or LL groups in the whole-genome resequencing data; iii) located at UTR or CDS regions; or iv) well suited for designing primers for time-of-flight (TOF) mass spectrometry.

A total of 228 female pigs, including 188 Landrace pigs and 40 large white pigs, were bred at Tianjin Farmer Farm Industry Technology Development Co., Ltd. (Ninghe, China) under standard conditions. Age, weight, and backfat thickness between the 3rd and 4th last ribs were determined. DNA samples were extracted from the ear tissues of the 228 pigs using the TIANamp Genomic DNA Kit (Tiangen Biotech). DNA samples were used to distinguish the genotypes by TOF mass spectrometry, which was completed at Generay Biotech (Shanghai, China).

To further explore the extent of linkage disequilibrium (LD) between each pair of SNPs in each gene, haplotypes were inferred for each individual. Measure of pairwise LD for all SNPs in one gene was performed using Haploview 4.2 software^47^. LD blocks were generated with genotype data using the LD coefficient (D’). Haplotype blocks within these SNPs were later used to test their associations with backfat thickness in subsequent analyses.

The single marker analysis method using the PROC GLM procedure in the SAS v8.1 software package (SAS Institute Inc., Cary, NC) was employed to analyse the association between genotypes and backfat thickness. The mixed model with the fixed effects was: *Y*_*ij*_ = *μ* + *G*_*i*_ + *B*_*j*_ + *e*_*ji*_, where *Y*_*ij*_ is the ij^th^ trait observation value; μ is the mean; *Gi* is the effect of the i^th^ genotypes; *B*_*j*_ is the effect of j^th^ breeding; and *e*_*ji*_ is the random residual that corresponds to the trait observation value. When marker-significant associations with traits reached *p* < 0.05, significant associations with backfat thickness were identified, and the association of the markers and backfat thickness was then analysed by linear regression analysis using SAS v8.1.

## Additional Information

**How to cite this article**: Xing, K. *et al.* Identification of genes for controlling swine adipose deposition by integrating transcriptome, whole genome resequencing, and quantitative trait loci data. *Sci. Rep.* **6**, 23219; doi: 10.1038/srep23219 (2016).

## Supplementary Material

Supplementary Information

## Figures and Tables

**Figure 1:**
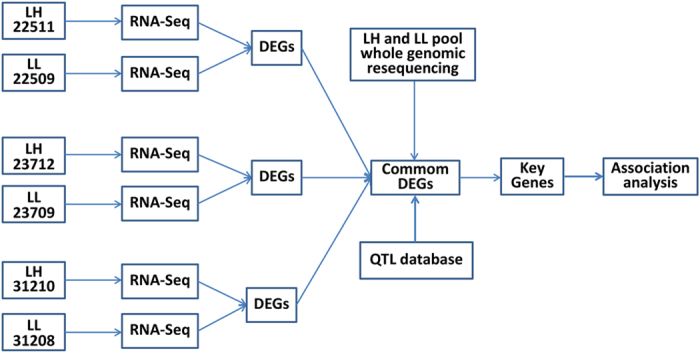
Experimental design. LH: The group of Landrace pigs with higher backfat thickness including individuals 22511, 23712, and 31210; LL: The group of Landrace pigs with lower backfat thickness including individuals 22509, 23709, and 31208. Individuals 22511 and 22509, 23712 and 23709, and 31210 and 31208 represent the three pairs of full-sibling pigs showing extremes of backfat thickness.

**Table 1:** Phenotype information related to fat deposition of three full-sibling Landrace pigs.

Individual ID	Backfat thickness oflive(mm)	Backfat thickness oflive after adjust(mm)	Backfat thicknessof carcass(mm)	Kidney fat(kg)
22511	9.7	10.94	14	0.5
22509	3.8	4.74	7.2	0.25
23712	8.8	9.83	13.1	0.6
23709	3.8	4.59	7.1	0.4
31210	7.6	8.85	13	0.6
31208	3.8	4.04	7.2	0.3

LH: Landrace pig group with higher backfat thickness; LL: Landrace pig group with lower backfat thickness; individuals 22511, 23712, and 31210 are in group LH, while 22509, 23709, and 31208 are in group LL; 22511/22509, 23712/23709, and 31210/31208 are the three full-sibling pairs of pigs.

**Table 2:** Pathway enrichment analysis of DEGs.

Expression level	Pathway	P value	Gene name
UP	Pyruvate metabolism	5.56E–07	*LDHA ME1 PDHB DLATALDH2 LDHD*
	Oxidative phosphorylation	1.33E–05	*COX6B NDUFB8 COX5BUQCRH SDHC PINK1UQCRFS1 UQCRQ*
	Glycolysis/Gluconeogenesis	1.09E–04	*LDHA PGK1 PDHB DLATALDH2*
	Cardiac muscle contraction	3.04E–04	*COX5B UQCRH UQCRFS1UQCRQ COX6B*
	Citrate cycle (TCA cycle)	7.70E–03	*SDHC PDHB DLAT*
Down	TGF-beta signaling pathway	9.64E–03	*ID1 ID2 COMP*

LH: Landrace pig group with higher backfat thickness; LL: Landrace pig group with lower backfat thickness. The threshold of significance was 0.05.

**Table 3:** Genetic variations in LH and LL groups and shared groups.

Variation Type	Specific LH		Specific LL		LH and LL	
	SNP	Indel	SNP	Indel	SNP	Indel
All	801,211	135,149	682,219	116,233	1,801,636	56,460
Intergenic	598,300	102,188	512,803	28,524	1,385,519	44,249
Intergenic(Upstream w/5-kb)	39,917	6,311	33,480	4,894	77,607	2,371
Intergenic(Downstream w/5-kb)	39,681	6,361	33,475	5,815	78,816	2,446
Intronic	212,641	34,971	178,117	28,524	439,062	13,051
3′UTR	3,187	582	2,396	449	5,866	178
5′UTR	508	67	409	54	645	16
Splice site	46	107	27	84	117	51
Splice site acceptor	22	54	16	43	51	22
Splice site donor	24	53	11	41	66	29
Start-gained	94	0	51	1	107	0
Start-loss	1	0	4	0	6	0
Stop-gained	23	0	17	0	35	0
Start-loss	3	0	0	0	11	0
Synonymous	3,052	NA	2,681	NA	5,213	NA
Nonsynonymous	1,969	NA	1,656	NA	4,151	NA
Frameshift indels	NA	288	NA	200	NA	114

LH: Landrace pig group with higher backfat thickness; LL: Landrace pig group with lower backfat thickness. Specific LH: genetic variants that were only detected in LH; Specific LL: genetic variants that were only detected in LL.

**Table 4:** Effects of *ME1* polymorphisms on adjusted backfat thickness.

Type	Position	Gene	Genotype	Number ofindividuals	Backfat thickness(mm)
SNP	Chr1-93240107	*ME1*	AA	16	10.28 ± 0.41^a^
			CA	91	11.56 ± 0.19^b^
			CC	119	11.37 ± 0.19^b^
SNP	Chr1-93240683	*ME1*	GG	120	11.37 ± 0.19^b^
			GT	90	11.26 ± 0.19^b^
			TT	16	10.28 ± 0.41^a^
haplotype	Chr1-93240107- Chr1-93240683	*ME1*	H1H1	16	10.28 ± 0.41^a^
			H1H2	90	11.56 ± 0.19^b^
			H2H2	118	11.37 ± 0.19^b^

Backfat thickness: LSM (Least Squares Means) ± SE (Standard Error). H1: H1:AT haplotype; H2: H2:CG haplotype. Superscript lowercase letters within the same type represent different means within a column at *p* < 0.05.
