# Supplementary material for: Identification of genes for controlling swine adipose deposition by integrating transcriptome, whole-genome resequencing, and quantitative trait loci data
Source: Sci Rep. 2016 Mar 21;6:23219. doi: 10.1038/srep23219 (PMC4800386; doi:10.1038/srep23219)
Supplement: Supplementary Information [file srep23219-s1.pdf]

Identification of genes for controlling swine adipose deposition by integrating transcriptome, whole genome resequencing, and quantitative trait loci data

Kai Xing[1], Feng Zhu[1], ShaoKang Chen[2], LiWei Zhai[1], Zhen Tan[1], YangYang Sun[1], ZhuoCheng Hou[1]* & ChuDuan Wang[1]*

[1]National Engineering Laboratory for Animal Breeding and MOA Key Laboratory of Animal Genetics and Breeding, Department of Animal Genetics and Breeding, China Agricultural University, Beijing 100193, China

[2]Beijing General Station of Animal Husbandry, Beijing 100125, China

* Corresponding authors

ZH, zchou@cau.edu.cn

CW, wangchuduan@qq.com

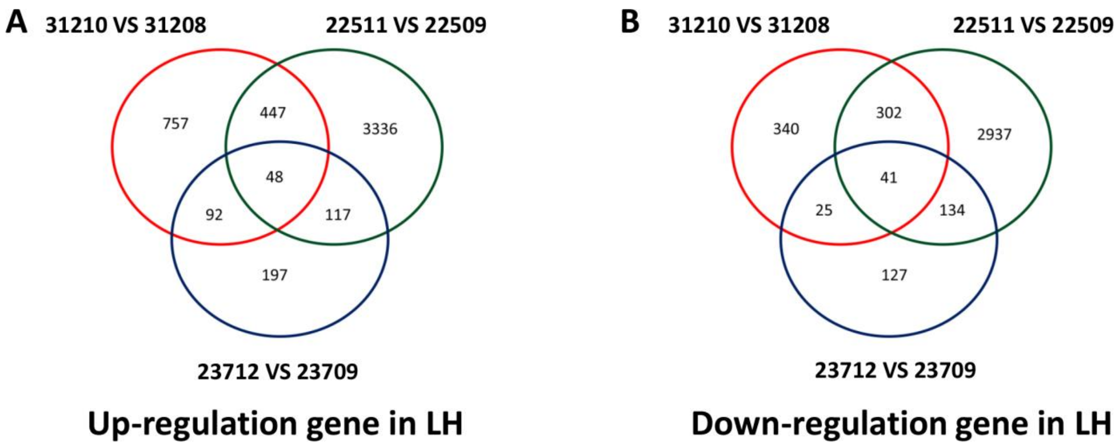

Supplementary Figure S1. Venn diagrams showing DEGs among each pair of pigs. Analysis of similarities in DEGs between each pair of pigs. (**A**) Genes significantly up-regulated in each comparison. (**B**) Genes significantly down-regulated in each comparison.

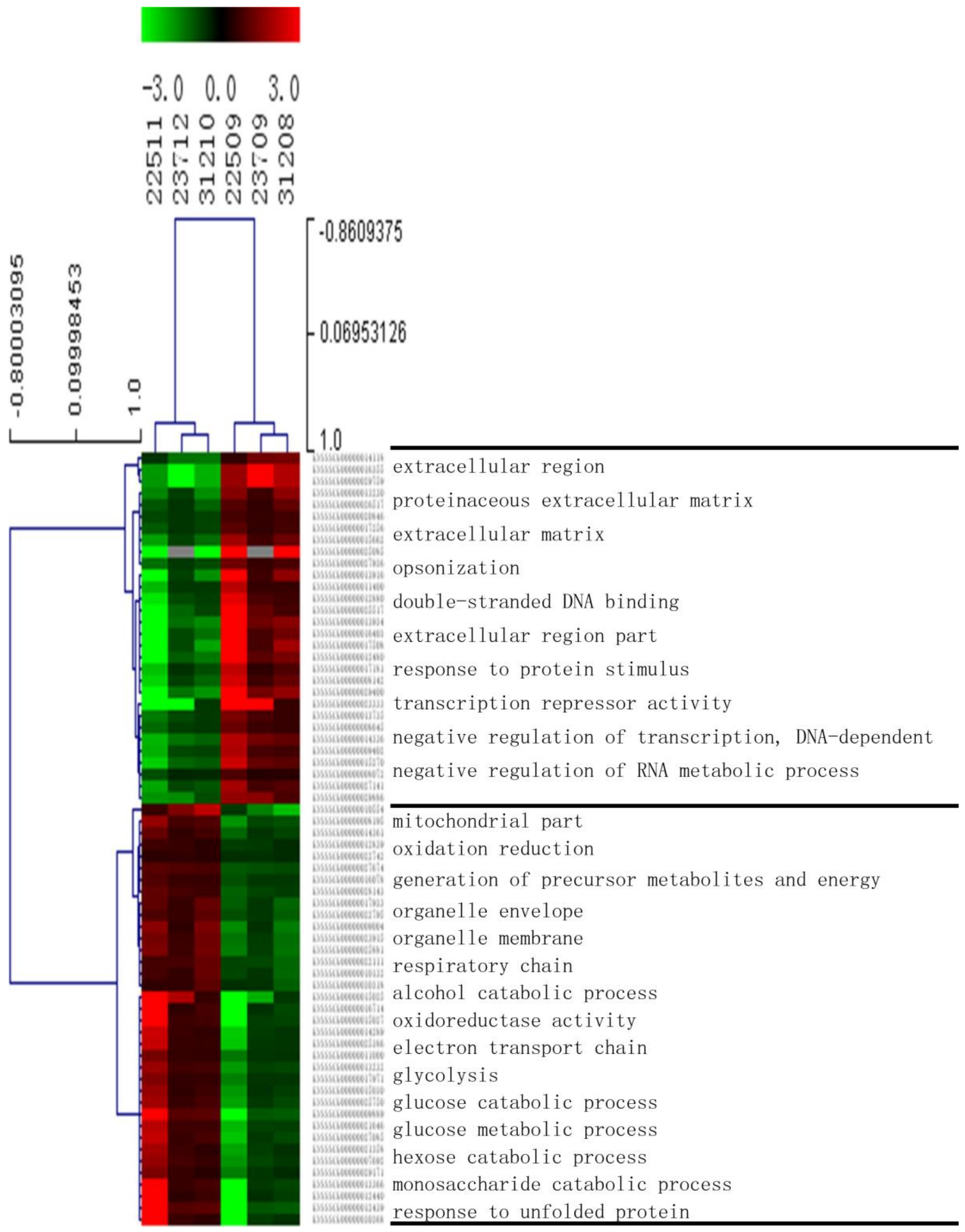

Supplementary Figure S2. Heat map and cluster analysis of common DEGs. Heat map showing fold-changes of DEGs in six samples (three in each of LH and LL groups). Genes significantly up-regulated are shown in red and those significantly down-regulated in green. Significant GO terms in DAVID are listed as threshold values ≤0.05.

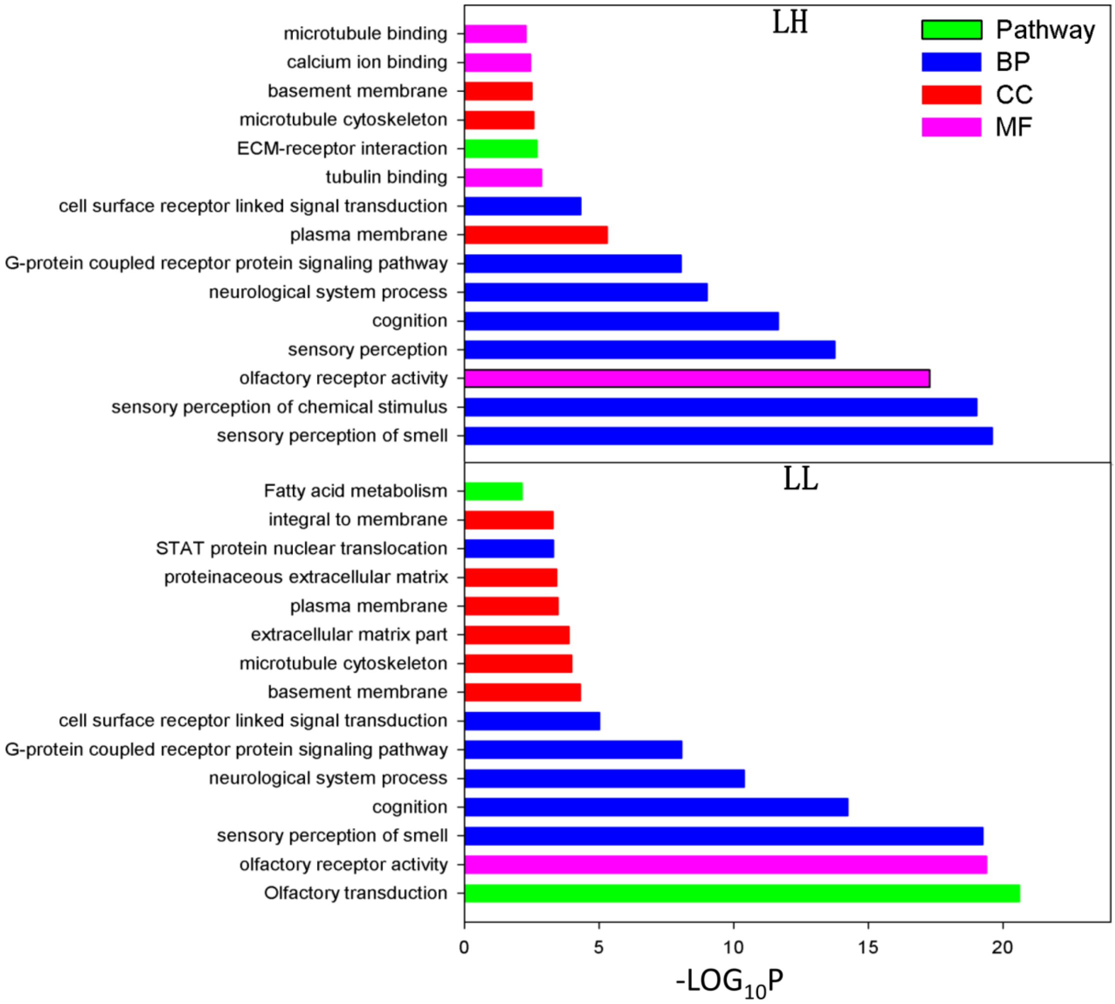

Supplementary Figure S3. Pathways and GO processes enriched in DAVID for genes with non-synonymous/coding indels. Genes with NS or CI were analysed using the DAVID functional annotation tool in LH and LL groups, respectively. The most significant pathways and GO terms are shown. x-axis represents $-\log_{10}$P.

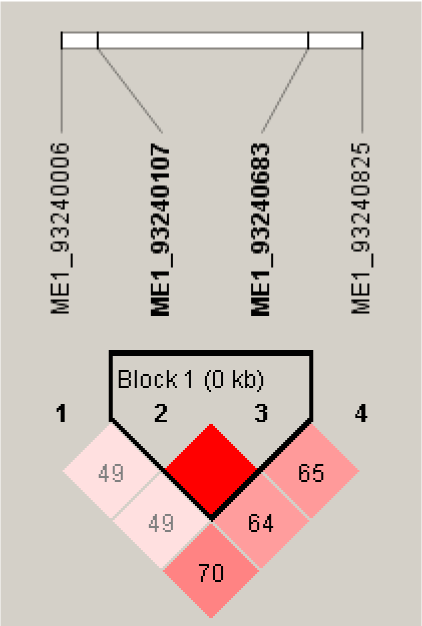

Supplementary Figure S4. Linkage disequilibrium plot for the four *ME1* SNPs. Two *ME1* SNPs showing significant association with backfat thickness make up one block.

Supplementary Table S1. Summary of RNA-seq reads.

Sample ID	Total reads (Million)	Mapping rate (%)	CDS exone(%)	5'UTR (%)	3'UTR (%)	Intron (%)
**22509**	38.55	78.37	69.91	2.10	13.56	5.80
**22511**	40.08	73.12	74.66	3.11	11.02	6.70
**23709**	38.03	81.37	73.73	1.74	11.30	5.57
**23712**	38.57	80.77	75.45	1.89	10.64	5.10
**31208**	37.19	80.06	70.70	1.99	12.22	6.95
**31210**	39.69	78.40	75.44	2.13	11.52	3.85

Ensembl v67 was used for reference genome annotation to classify mapping tags into different regions. The ratio of the tags mapping onto the subregion of the gene was calculated as the tags within each region divided by the total tags in the whole genome.

Supplementary Table S2. DEGs related to lipid synthesis, transport, and metabolism.

Gene symbol	Ensembl Gene ID	23711vs23709			22511vs22509			31210vs31208			Fat process
		M	D	P	M	D	P	M	D	P	
ALDH2	ENSSSCG00000009889	0.99	44.69	0.90	3.25	222.65	1.00	1.06	39.15	0.92	Fatty acid degradation
ATP5J2	ENSSSCG00000029171	0.61	222.15	0.83	1.49	702.00	0.96	0.48	244.88	0.80	fatty acid synthesis
CDKN1A	ENSSSCG00000001565	0.89	26.74	0.87	1.21	60.54	0.94	1.28	58.21	0.95	adipocyte differentiation
COMP	ENSSSCG00000013916	−0.81	25.56	0.86	−7.58	24.44	0.99	−1.72	18.59	0.96	Lipid metabolism
COMT	ENSSSCG00000010132	0.58	43.86	0.82	0.83	28.09	0.86	1.19	86.67	0.94	Lipid metabolism
COX5B	ENSSSCG00000008195	0.80	496.54	0.87	1.61	199.51	0.96	0.93	893.91	0.90	fatty acid oxidation
COX6B	ENSSSCG00000002907	0.53	295.38	0.81	2.16	1569.91	0.99	0.90	634.74	0.89	fatty acid synthesis
CPT1A	ENSSSCG00000012880	−0.93	18.31	0.88	−2.73	19.66	0.99	−0.78	14.90	0.86	Lipid metabolism
CYP21A2	ENSSSCG00000001428	−1.09	50.77	0.93	−2.00	41.08	0.98	−0.79	37.84	0.87	Lipid metabolism
DLAT	ENSSSCG00000015030	0.56	16.23	0.80	1.80	39.16	0.96	0.70	24.06	0.84	Fatty acid biosynthesis
HSPA1A	ENSSSCG00000030368	0.67	163.65	0.84	3.33	1272.59	1.00	0.55	64.82	0.82	Lipid metabolism
ID1	ENSSSCG00000007227	−0.88	75.49	0.88	−1.07	68.47	0.92	−0.92	85.64	0.90	lipid metabolism and insulin sensitivity
ID2	ENSSSCG00000008645	−0.77	21.47	0.85	−1.26	25.78	0.94	−0.69	21.65	0.84	adipose differentiation and fatty acid synthesis
KLF2	ENSSSCG00000005437	−1.05	44.30	0.92	−1.20	30.87	0.94	−0.52	16.90	0.80	adipose differentiation
LDHA	ENSSSCG00000013366	0.65	50.80	0.84	7.22	11118.73	1.00	0.79	75.97	0.87	fatty acid synthesis
LDHD	ENSSSCG00000002712	0.96	9.82	0.87	2.81	20.84	0.99	1.54	16.38	0.95	oxidation reduction
LPAR6	ENSSSCG00000009402	0.55	131.77	0.81	−2.08	14.12	0.97	−0.83	19.47	0.87	preadipocyte differentiation
ME1	ENSSSCG00000004454	0.55	120.27	0.81	0.61	13.00	0.81	2.98	603.83	1.00	Fatty acid biosynthesis
NDUFB8	ENSSSCG00000006166	0.54	64.92	0.81	2.81	20.84	0.99	0.68	114.15	0.85	fatty acid synthesis
PDHB	ENSSSCG00000025881	0.69	78.77	0.85	1.47	74.46	0.95	1.31	142.93	0.95	lipid biosynthesis and adipocyte

											maturation
PRDX6	ENSSSCG00000022742	−0.90	14.27	0.87	0.56	81.76	0.81	0.49	119.26	0.80	fatty acid metabolism
SCD	ENSSSCG00000010554	1.45	6781.47	0.96	0.68	88.53	0.84	2.21	7873.97	0.99	Fatty acid biosynthesis
SDHC	ENSSSCG00000030318	0.56	115.66	0.82	0.59	90.07	0.82	1.09	301.00	0.93	energy metabolism
SFRP2	ENSSSCG00000009004	0.55	31.46	0.81	1.63	50.53	0.96	1.44	130.44	0.96	insulin sensitivity
SLC2A4	ENSSSCG00000023915	0.67	34.74	0.84	1.37	65.93	0.95	1.27	83.49	0.95	Fatty acid biosynthesis
UQCRH	ENSSSCG00000003903	1.96	656.03	0.98	1.96	656.03	0.98	0.82	368.16	0.88	fatty acid synthesis
VIPR2	ENSSSCG00000016403	−0.90	6.75	0.83	−4.76	10.21	0.98	−1.37	7.12	0.90	fatty acid oxidation

M: contrasting fold-change differences; D: absolute expression differences

Supplementary Table S3. Common traits compared with the QTL database and previous studies.

Gene symbol	Traits related to fat deposition in overlap QTLs	Overlap previous RNA-seq results
SDHC	Intramuscular fat; Subcutaneous fat; Side fat; Fat percentage; intestinal fat; backfat; Adipocyte diameter	NA
MTCH2	Backfat; subcutaneous fat	NA
LDHA	Backfat; Fat-cuts percentage; Fat to meat ratio; subcutaneous fat	NA
CYCS	Backfat	Reference [14]
LOC100524873	Backfat; Fat area; Intramuscular fat	NA
LDHD	Adipocyte diameter; Backfat; Fat area; Intramuscular fat; Fat to meat ratio	NA
UQCRQ	Backfat; Leaf fat; Ham fat; Intramuscular fat	NA
TMEM245	Backfat; Fat to meat ratio; Ham fat; Adipocyte diameter; Fat-cuts	NA
SNTA1	Backfat	NA
ROMO1	Backfat; intestinal fat; Fat area	NA
FCN2	Backfat; Leaf fat weigh	Reference [11,14]
FNDC1	Backfat; subcutaneous fat	NA
NEGR1	Fat to meat ratio; Fat area; Intramuscular fat; Fat weight (total); Backfat	NA
CYGB	Backfat	NA
CYP21A2	backfat; Leaf fat; Adipocyte diameter; Fat protein content; Fat area; subcutaneous fat	NA
C4	backfat; Leaf fat weight; Adipocyte diameter; Fat protein content; Fat area	Reference [12,14]
KLF4	backfat; Ham fat; Fat to meat ratio; Adipocyte diameter; Leaf fat weight; Fat-cuts percentage	Reference [14]
S100A4	Intramuscular fat content; intestinal fat; Abdominal fat; Backfat	Reference [11]
ASPN	Side fat	Reference [11,14]
ID1	Backfat	Reference [14,20]
SCD	NA	Reference [20]
HSP70.2	NA	Reference [20]
STAC2	NA	Reference [20]
CRYAB	NA	Reference [20]
FMOD	NA	Reference [12]
LYVE1	NA	Reference [12]
FAM180B	NA	Reference [12]
TNMD	NA	Reference [11,14,20]
RBP5	NA	Reference [14]

PINK1	NA	Reference [14]
SFRP2	NA	Reference [14]
MATN4	NA	Reference [14,20]
C3H2orf40	NA	Reference [14]
ABCA6	NA	Reference [14]
HSPE1	NA	Reference [14]
SLC2A4	NA	Reference [14]
ID2	NA	Reference [14]
ME1	NA	Reference [14,20]
CHAC1	NA	Reference [11,20]
DLAT	NA	Reference [11]
FNDC1	NA	Reference [11]

Supplementary Table S4. Genetic variations in DEGs detected only in LH and LL groups.

Variation Type	Specific LH		Specific LL	
	SNP	Indel	SNP	Indel
Intergenic(Upstream w/5-kb)	130	25	130	38
Intergenic(Downstream w/5-kb)	98	22	94	20
Intronic	568	142	444	132
Synonymous	14	NA	6	NA
Nonsynonymous	7	NA	4	NA
3’UTR	5	3	26	4
5’UTR	2	1	0	0

Specific LH: genetic variants only detected in LH; Specific LL: genetic variants only detected in LL.

Supplementary Table S5. Important SNPs in key DEGs effecting backfat thickness.

SNP	Gene	Genotype frequency/%			Gene frequency/%		*p*-value
SNP1	*SCD*	CC/1	TC/7	TT/91	C/5	T/95	0.96
SNP2	*SCD*	AA/70	CA/18	CC/12	A/79	C/21	0.41
SNP3	*ME1*	CC/45	TC/2	TT/53	C/46	T/54	0.50
SNP4	*ME1*	AA/7	CA/40	CC/53	A/27	C/73	0.02*
SNP5	*ME1*	GG/53	GT/40	TT/7	G73	T/27	0.02*
SNP6	*ME1*	CC/38	TC/6	TT57	C/40	60	0.97
SNP9	*FASN*	AA/8	GA/54	GG/38	A/35	G/65	0.33
SNP10	*FASN*	CC/99	TC/1	TT/0	C/99.5	A/0.5	0.79
SNP11	*FASN*	AA/8	GA/54	GG/38	A/35	G/65	0.37
SNP12	*FASN*	AA/37	GA/56	GG/7	A/64	G/35	0.24
SNP14	*PCK1*	CC/92	TC/7	TT1	C/96	T/4	0.63

p-value: associated with SNP effect on backfat thickness; * indicates significant (<0.05).

Supplementary Table S6. Information about selected SNPs.

SNP	DEG	Position	variation type	Wide type	Mutant type	group
**SNP1**	*SCD*	Chr14-120964624	5'UTR	C	T	LL
**SNP2**	*SCD*	Chr14-120979878	3'UTR	C	A	LL
**SNP3**	*ME1*	Chr1-93240006	3'UTR	C	T	LL
**SNP4**	*ME1*	Chr1-93240107	3'UTR	A	C	LL
**SNP5**	*ME1*	Chr1-93240683	3'UTR	T	G	LL
**SNP6**	*ME1*	Chr1-93240825	3'UTR	C	T	LL
**SNP7**	*FASN*	Chr12-1034641	5'UTR	A	C	LL
**SNP8**	*FASN*	Chr12-1023517	NSC	A	C	LH
**SNP9**	*FASN*	Chr12-1025096	NSC	G	A	LH
**SNP10**	*FASN*	Chr12-1025252	NSC	T	C	LH
**SNP11**	*FASN*	Chr12-1030120	NSC	G	A	LH
**SNP12**	*FASN*	Chr12-1031763	NSC	A	G	LH
**SNP13**	*CRYAB*	Chr9-44432848	5'UTR	A	G	LH
**SNP14**	*PCK1*	Chr17-65094895	5'UTR	C	T	LL

DEG: differential expression between LH and LL groups; NSC: non-synonymous coding; UTR: untranslated region; group: group in which each SNP was detected.
